# A New Method for Fitting Current–Voltage Curves of Planar Heterojunction Perovskite Solar Cells

**DOI:** 10.1007/s40820-017-0159-z

**Published:** 2017-10-13

**Authors:** Peizhe Liao, Xiaojuan Zhao, Guolong Li, Yan Shen, Mingkui Wang

**Affiliations:** 1 Wuhan National Laboratory for Optoelectronics, Huazhong University of Science and Technology, Luoyu Road 1037, Wuhan, 430074 People’s Republic of China; 2 Ningxia University, Helan Mountain Road 489, Yinchuan, 750021 People’s Republic of China

**Keywords:** Dark current, Device simulation, Junction property, Perovskite, Solar cell

## Abstract

Herein we propose a new equivalent circuit including double heterojunctions in series to simulate the current–voltage characteristic of P–I–N planar structure perovskite solar cells. This new method can theoretically solve the dilemma of the parameter diode ideal factor being larger than 2 from an ideal single heterojunction equivalent circuit, which usually is in the range from 1 to 2. The diode ideal factor reflects PN junction quality, which influences the recombination at electron transport layer/perovskite and perovskite/hole transport layer interface. Based on the double PN junction equivalent circuit, we can also simulate the dark current–voltage curve for analyzing recombination current (Shockley–Read–Hall recombination) and diffusion current (including direct recombination), and thus carrier recombination and transportation characteristics. This new model offers an efficacious and simple method to investigate interfaces condition, film quality of perovskite absorbing layer and performance of transport layer, helping us further improve the device efficiency and analyze the working mechanism.

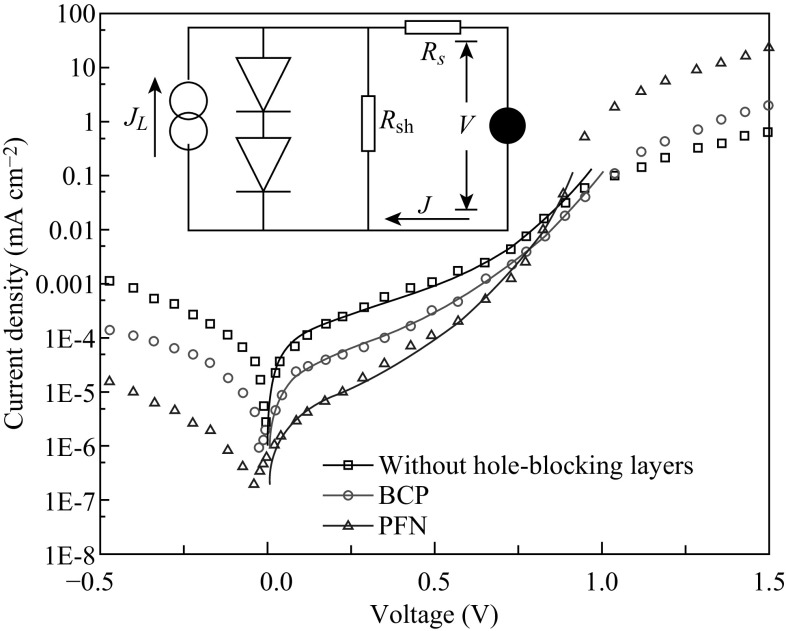

## Highlights

- A universal and simple method to analyze current-voltage curves of planar heterojunction perovskite solar cells is proposed.
- The new method theoretically solves the dilemma of the parameter diode ideal factor being larger than 2.
- The dark current fitted with the new method helps to analyze physical processes of perovskite solar cells.

## Introduction

The photoelectric effect converts solar energy into electricity, which is one of the promising ways to solve the global energy crisis and environmental pollution. Due to the excellent light absorption and carrier transportation characteristics, perovskite-type semiconductors with a general ABX_3_ formula have attracted intensive interest in recent years [1]. The power conversion efficiency (PCE) of perovskite solar cell (PSC) has a rapid growth from 3.8% in 2009 to 22.1% in 2016 [2, 3]. Despite this, it is important to understand the carrier transport mechanism of PSCs, while it is a good way to fit current–voltage (*J*–*V*) curves. Hence the *J*–*V* curves for silicon solar cells and thin-film solar cells have been fitted to analyze the working mechanism and performance of solar cells [4–6]. Considering the absence of specific equivalent circuit and fitting formula for P–I–N model, an ideal single PN junction circuit has been built to simulate the *J*–*V* characteristic of various PSCs [7]. By fitting *J*–*V* curves under light and in dark, three parameters including series resistance (*R*_s_), diode ideal factor (m) and reverse saturation current (*J*_0_) can be obtained. Compared to the reverse saturation current of conventional semiconductor diodes (such as CdTe, GIGS), the parameter *J*_0_ of PSC is relatively low, which explains its smaller bandgap voltage loss (~ 0.4 eV) [8]. Therefore, it is helpful to improve the efficiency of PSC by understanding the two parameters of *R*_s_ and *J*_0_ [9]. Furthermore, the parameter m has been utilized as an indication of the heterojunction solar cell [7].

**Fig. 1:**
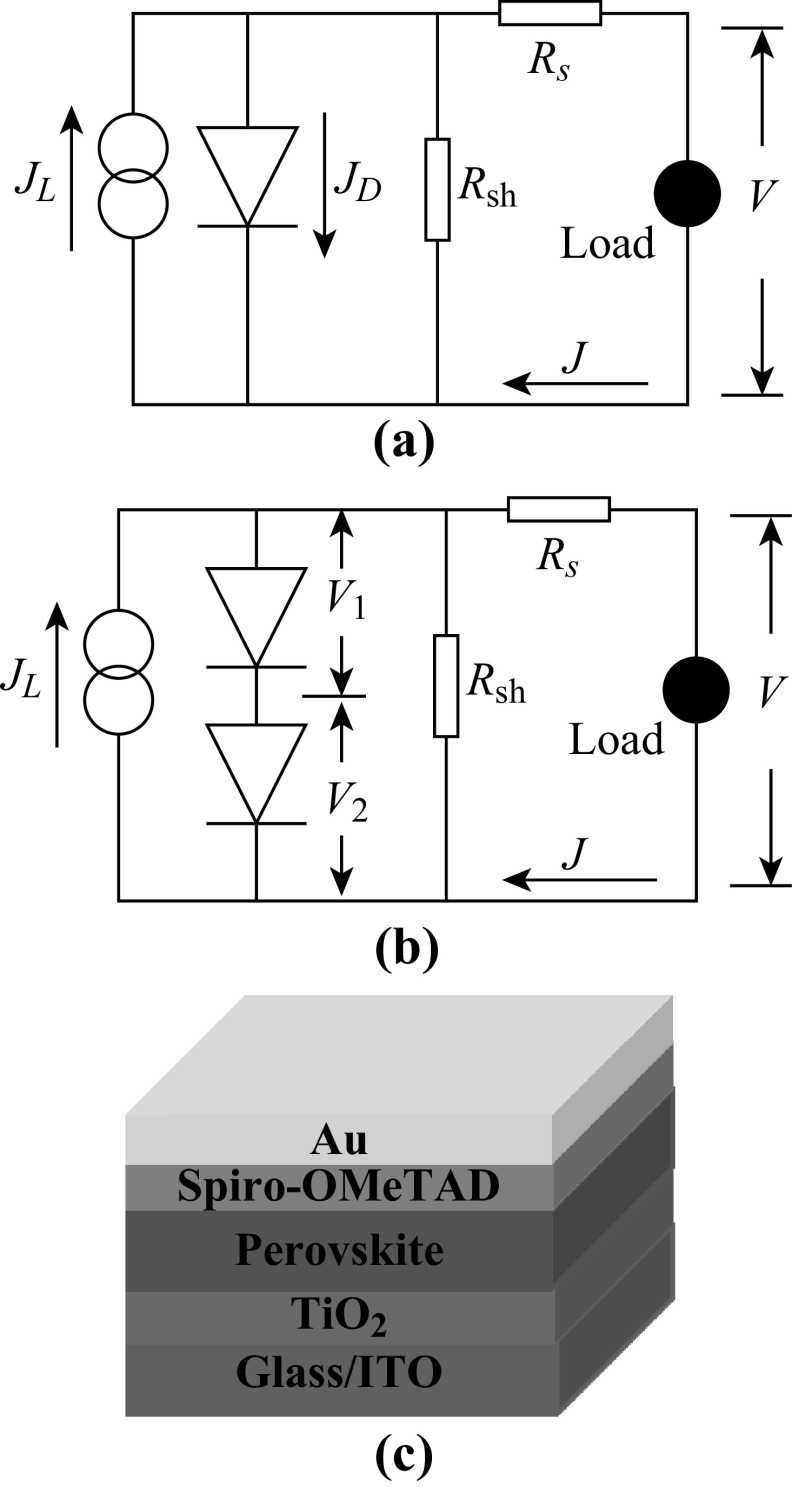
**a** Single PN junction model for PSCs and **b** new double PN junction model improved for planar perovskite solar cells with *J*_L_ (the light induced current), *J*_D_ (the dark current or the forward current of PN junction diode under the sunlight), *R*_s_ (the series resistance), *R*_sh_ (shunt resistance, a fictional parameter to represent the size of leakage current), *J* (output current of the cell) and *V* (voltage flowing through the external load). **c** Planar heterojunction perovskite solar cells with TiO_2_/CH_3_NH_3_PbI_3-x_Cl_x_/Sprio-OMeTAD/Au device architecture. (Color figure online)

To date, planar-structured PSCs have been developing rapidly due to their various advantages of simple device structure, low-temperature processable fabrication and so on [10]. Nevertheless, in planar PSCs, the value of m obtained by single PN junction modeling does not fill in the theoretical expectation, indicating that the heterojunction property in planar PSCs further discussion [11, 12]. Generally, for a single heterojunction model, the ideal factor approaches to 1 when the carrier diffusion in the neutral region of semiconductors dominates the diode current through a PN junction. On the other hand, the ideal factor approaches to 2 when the diode current is dominated by carrier indirect recombination in depleted space-charge region [9]. Theoretically, the smaller value of m reflects the less carrier recombination induced by the interface defect state. In most cases, both diffusion and composite currents exist simultaneously, and therefore, the parameter m is in the range of 1–2. Interestingly, we note that, as shown in Table 1, most of the calculation results are larger than 2. Hence a single PN junction model (Fig. 1a) is not suitable to planar heterojunction PSCs [13, 14].

**Table 1:** Calculated ideal factor (*m*) on planar heterojunction PSCs based on single PN junction model reported in the literatures

Device architecture	Ideal factor (*m*)	References
ITO/PEDOT:PSS/CH_3_NH_3_PbI_3-x_Cl_x_/PCBM/PFN/Al	2.3	[16]
FTO/CH_3_NH_3_PbI_3-x_Cl_x_/Spiro-OMeTAD/Au	3.39	[38]
Mg-ZnO/CH_3_NH_3_PbI_3-x_Cl_x_/Spiro-OMeTAD/Au	2.6–3.0	[39]
ITO/PEDOT:PSS/Perovskite/PCBM/BCP/Ag	2.2	[40]
ITO/PEDOT:PSS/CH_3_NH_3_PbI_3_/C_60_/Al	3.51	[41]
FTO/Mg-TiO_2_/Perovskite/Spiro-OMeTAD/Au	2.1	[42]
FTO/TiO_2_/C_60_/(FA)_x_(MA)_1-x_PbI_3_/Spiro-OMeTAD/Au	2.6–2.92	[43]

In this work, we present a new equivalent circuit to investigate the heterojunction property for planar PSCs (in light and dark). Based on the new double heterojunction circuit, we found that smaller value of m reflects better PN junction quality in PSCs. Moreover, carrier recombination and transportation characteristics can be further explored by fitting *J*–*V* curve in dark with the new model for describing these important processes in efficient PSC devices.

## Theoretical Background

Firstly, the rectification characteristic of heterojunction solar cell can be typically described by the Shockley diode equation (Eq. 1) [14],

1 $$J_{\text{D}} = J_{0} \left\{ {e^{{\frac{qV}{mKT}}} - 1} \right\}$$

where *J*_D_ is the dark current, *V* is the applied voltage, *J*_0_ is the reverse saturation current density, *q* is the elementary charge, *m* is the ideal factor of a heterojunction, *K* is the Boltzmann constant, *T* is the absolute temperature. Under the ideal condition of sunlight, photocurrent can be added into Eq. 1:

2 $$J = J_{\text{ph}} - J_{0} \left\{ {e^{{\frac{qV}{mKT}}} - 1} \right\}$$

where *J*_ph_ is the photocurrent. In fact, output current (*J*) in Eq. 2 is limited by internal resistance and leakage current in PSCs. Figure 1a presents ideal circuit model with a single PN junction, from which the *J*–*V* curve (in light) of heterojunction PSC can be further described with Eq. 3 [15],

3 $$J = J_{\text{ph}} - J_{0} \left\{ {e^{{\frac{{q\left( {V + JR_{\text{s}} } \right)}}{mKT}}} - 1} \right\} - \frac{{V + JR_{\text{s}} }}{{R_{\text{sh}} }}$$

where *R*_s_ and *R*_sh_ are the series and shunt resistance, respectively. Under the circumstances, *R*_s_, *R*_sh_, *J*_0_ and m can be numerically obtained by simulation the *J*–*V* curves (both in light and dark) of PSCs with Eq. 3. The *R*_s_ reflects the internal resistance, and *R*_sh_ is a fiction parameter to represent the leakage current. The value of *J*_0_ is directly related to the recombination rate, indicating the thermal emission rate of electrons from the valence band to the conduction band in light absorption layer [16], which also has an impact on the open-circuit voltage. Nevertheless, compared with *R*_s_ and *J*_0_, *m*, correlating with Shockley–Read–Hall recombination [6], is a rarely discussed parameter in PSCs when fitting *J*–*V* curves with using single PN junction model [17–20].

**Fig. 2:**
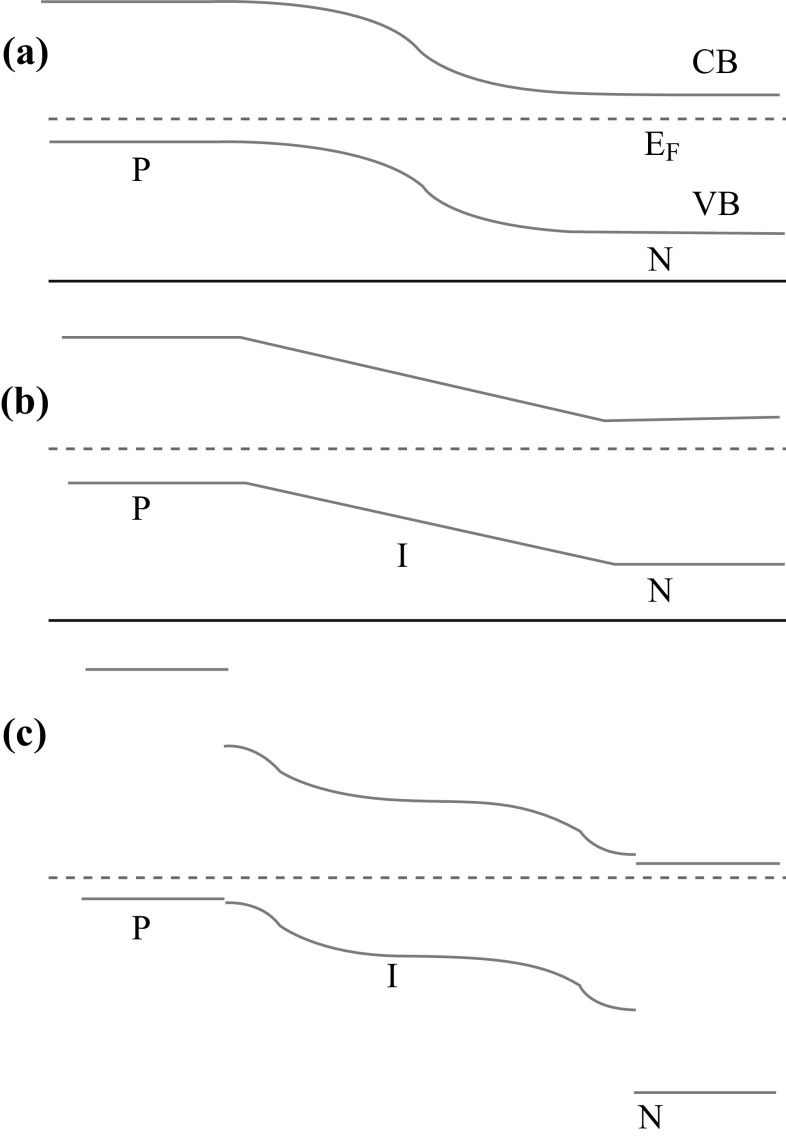
Energy band diagram of different PN junction photovoltaic devices. **a** A PN junction solar cell. **b** A P–I–N solar cell with homogeneous built-in electric field. **c** CH_3_NH_3_PbI_3-x_Cl_x_ perovskite-based cell with inhomogeneous built-in electric field. (Color figure online)

Compared to traditional P–I–N structure solar cells (‘a-Si: H’-like), inhomogeneous built-in field of PSCs results in different band structure (Fig. 2) [21, 22]. When the perovskite light absorption layer is sandwiched between n- and p-type charge selective contacts (Fig. 1c), two active junctions immediately form at the n-type electron transport layer (ETL) and the p-type hole transport layer (HTL) sides [22]. Therefore, we suggest two PN junctions in series for explaining planar heterojunction PSCs [23–27], rather than a single PN junction. Equation 4 is applied according to the equivalent circuit of double PN junction as shown in Fig. 1b:

4 $$V_{1} + V_{2} = V + JR_{\text{s}}$$

According to the characteristics of the series circuit, the current through the double PN junction should be identical (Eq. 5).

5 $$J_{01} \left\{ {e^{{\frac{{qV_{1} }}{{m_{1} KT}}}} - 1} \right\} = J_{02} \left\{ {e^{{\frac{{qV_{2} }}{{m_{2} KT}}}} - 1} \right\}$$

where m_1_, *V*_1_, *J*_01_, *m*_2_, *V*_2_, *J*_02_ are diode ideality factor, voltage and reverse saturation current of ETL/perovskite and perovskite/HTL two PN junctions, respectively.

In this study, perovskite absorption layer acts as intrinsic semiconductor, which is fully depleted with highly doped P/N selective layers to form versatile PIN photovoltaics [28]. Considering the condition of *J*_01_∝*P*_n_ *D*_p_/L_p_, *J*_02_∝*N*_p_ *D*_n_/L_n_ and a similar carrier density for electrons and holes [29, 30], the value of *D*_p_/*L*_p_ can be approximately equal to *D*_n_/*L*_n_ [31]. Therefore, the difference of the calculated *J*_01_ and *J*_02_ values lies in the same magnitude in this case according to the derivation process (Eq. 6).

6 $$J_{01} \cong J_{02} = J_{0}$$

Then Eq. 3 can be further revised as Eq. 7:

7 $$J = J_{\text{ph}} - J_{01} \left\{ {e^{{\frac{{qV_{1} }}{{m_{1} KT}}}} - 1} \right\} - \frac{{V + JR_{\text{s}} }}{{R_{\text{sh}} }}$$

According to Eqs. 4–7, Eq. 8 and Eq. 9 can be inferred:

8 $$J = J_{\text{L}} - J_{0} \left\{ {e^{{\frac{{q\left( {V + JR_{\text{s}} } \right)}}{{\left( {m_{1} + m_{2} } \right)KT}}}} - 1} \right\} - \frac{{V + JR_{\text{s}} }}{{R_{\text{sh}} }}$$

9 $$\frac{{V_{1} }}{{m_{1} }} = \frac{{V_{2} }}{{m_{2} }} = \frac{{V + JR_{\text{s}} }}{{m_{1} + m_{2} }}$$

Equation 8 describes the *J*–*V* curve of planar PSCs under illumination. Since *m* in Eq. 3 includes the contribution from the double junctions, the sum of *m*_1_ and *m*_2_ can be in the range of 2–4. In short, the calculation results of *m* (~ 2–4) in Table 1 confirm the suitability of the proposed double heterojunction equivalent circuit for the planar PSC devices, with which carrier transportation (including direct recombination) and recombination (Shockley–Read–Hall recombination) processes can be preciously described.

## Results and Discussion

**Fig. 3:**
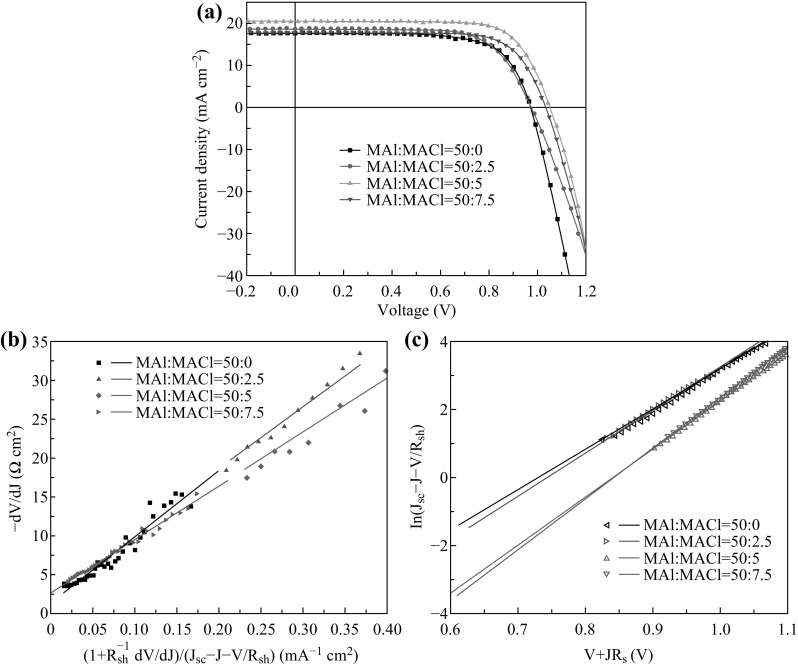
**a** Current–voltage curve for planar perovskite solar cells using TiO_2_/CH_3_NH_3_PbI_3-x_Cl_x_/Spiro-OMeTAD/Au architecture, the perovskite films prepared by mixing with different concentrations of Cl ions. The measurements are carried out under 100 mW cm^−2^. **b** Plots of $- {\text{d}}V/{\text{d}}J \quad {\text{versus}}\quad \left( {\frac{{1 + R_{\text{sh}}^{ - 1} \frac{{{\text{d}}V}}{{{\text{d}}J}}}}{{J_{\text{sc}} - J - V/R_{\text{sh}} }}} \right)$ (symbols) and the linear fitted curve (solid lines). **c** Plots of ln(*J*_sc_ − *J* − *V*/*R*_sh_) versus *V* + *JR*_s_ (symbols) and the linear fitted curve (solid lines). (Color figure online)

In order to elucidate the effect of *m* on planar PSCs, we fabricated CH_3_NH_3_PbI_3_-based planar PSC devices with structure of ITO/TiO_2_/CH_3_NH_3_PbI_3-x_Cl_x_/Spiro-OMeTAD/Au using two-step deposition method [23], in which methylammonium chloride (MACl) was added to increase the perovskite films quality. Figure 3a shows the typical *J*–*V* characteristics of these devices under simulated sunlight at 100 mW cm^−2^ (AM 1.5G). Table 2 shows the photovoltaic parameters of devices with perovskite layers by varying MACl concentration.

**Table 2:** Photovoltaic performance of perovskite solar cells fabricated from doping varied MACl concentration

MAI:MACl	*J*_sc_ (mA cm^−2^)	*V*_oc_ (V)	FF (%)	PCE (%)
50:0	17.81	0.96	68.87	11.8
50:2.5	18.64	0.98	68.75	12.5
50:5	20.35	1.05	72.73	15.6
50:7.5	17.54	1.03	73.53	13.3

The addition of Cl^−^ in the perovskite film significantly improved the efficiency of planar PSCs. The maximum performance was obtained for doping the appropriate amount of MACl (MAI:MACl = 50:5), which resulted in highly efficient devices exhibiting short-circuit current (*J*_sc_) of 20.35 mA cm^−2^, open-circuit voltage (*V*_oc_) of 1.05 V, fill factor (FF) of 72.73%, corresponding to PCE of 15.6%. The keys to improve the performance of CH_3_NH_3_PbI_3-x_Cl_x_ devices were better crystallinity of perovskite film, pin hole-free coverage of the perovskite films and fewer interface defect states [32, 33].

We further fitted these *J*–*V* curves of devices in Fig. 3a to investigate the effect of doping Cl on perovskite layers by analyzing the parameters of *R*_sh_, *R*_s_, *m*_1_ + *m*_2_ and *J*_0_. *R*_sh_ can be calculated from the inverse of the slope of the *J*–*V* curves at 0 V [34]. The other three parameters (*R*_s_, *m*_1_ + *m*_2_, *J*_0_) can be obtained through deduction of Eq. 8 as follows,

10 $$- \frac{{{\text{d}}V}}{{{\text{d}}J}} = \frac{{\left( {m_{1} + m_{2} } \right)KT}}{q}\left( {\frac{{1 + R_{\text{sh}}^{ - 1} \frac{{{\text{d}}V}}{{{\text{d}}J}}}}{{J_{\text{sc}} - J - V/R_{\text{sh}} }}} \right) + R_{\text{s}}$$

11 $$\ln \left( {J_{\text{sc}} - J - V/R_{\text{sh}} } \right) = \frac{q}{{\left( {m_{1} + m_{2} } \right)KT}}\left( {V + JR_{\text{s}} } \right) + \ln J_{0}$$

*R*_s_ can be obtained by calculating the intercept of fitting curve of $- {\text{d}}V/{\text{d}}J\quad {\text{versus}}\quad \left( {\frac{{1 + R_{\text{sh}}^{ - 1} \frac{{{\text{d}}V}}{{{\text{d}}J}}}}{{J_{\text{sc}} - J - V/R_{\text{sh}} }}} \right)$ (Eq. 10) in Fig. 3b. Similarly, *J*_0_ can be obtained by fitting the curve of ln(*J*_sc_ − *J* − *V*/*R*_sh_) versus (*V* + *JR*_s_) (Eq. 11) in Fig. 3c. The value of (*m*_1_ + *m*_2_) can be simultaneously inferred from the slope of fitting curves (Eq. 11) in Fig. 3c. The calculation results of four parameters (*R*_sh_, *R*_s_, *m*_1_ + *m*_2_, *J*_0_) are shown in Table 3.

**Table 3:** *R*_sh_, *R*_s_, *m*_1_ + *m*_2_ and *J*_0_ derived from Fig. 3b, c

MAI:MACl	*R*_sh_ (Ω cm^2^)	*R*_s_ (Ω cm^2^)	*m*_1_ + *m*_2_	*J*_0_ (mA cm^−2^)
50:0	840	1.41	3.27	1.69 × 10^−4^
50:2.5	5050	2.72	3.07	8.23 × 10^−5^
50:5	4280	2.61	2.66	6.89 × 10^−6^
50:7.5	3400	2.71	2.64	4.10 × 10^−6^

As shown in Table 3, the shunt resistance *R*_sh_ dramatically increases from 840 to 5050, 4280, and 3400 Ω for addition of Cl ions (MAI:MACl from 50:0 to 50:7.5) in perovskite films. A larger *R*_sh_ indicates less leakage current. This could be related to less pinholes for perovskite layers with Cl^−^ than that without additives [32, 35]. It is significant that the ideal factor (*m*_1_ + *m*_2_) drastically decreases from 3.27 to 2.66 after adding Cl^−^ and then keeps similar value of 2.66. The value of ideal factor (*m*_1_ + *m*_2_) is 3.27, 3.07, 2.66, and 2.64 for corresponding devices, respectively, which further conforms the feasibility of the double PN junction model. Smaller value of *m*_1_ + *m*_2_ indicates better PN junction quality [6]. The reverse saturation current (*J*_0_) of corresponding devices was estimated to be 1.69 × 10^−4^, 8.23 × 10^−5^, 6.89 × 10^−6^ and 4.10 × 10^−6^ mA cm^−2^. The smaller *J*_0_ is a sign of substantially suppression of the thermal emission rate of electrons from the VB to the CB [16], resulting in higher output voltage. This is verified with the *V*_oc_ (being 1.05 V) of device with the suitable addition of Cl ions (MAI:MACl = 50:5) in perovskite layer, which is higher than devices without Cl. In short, the calculated *R*_sh_, *m*_1_ + *m*_2_ further indicates that larger short-circuit current (20.35 mA cm^−2^) for the device with Cl can be attributed to less carrier recombination and loss in ETL/perovskite and perovskite/HTL interfaces.

**Fig. 4:**
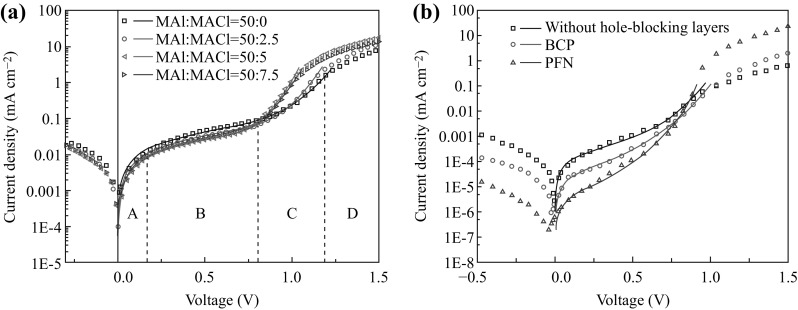
Plots of dark current of planar PSCs as well as fitting curve by using Eq. 13. **a** The device structure is shown in Fig. 1c, the perovskite films prepared by mixing with different concentrations of Cl ions. **b** The device structure is PEDOT:PSS/CH_3_NH_3_PbI_3-x_Cl_x_/PCBM/hole-blocking layer/Al, with the original data coming from the Ref. [8]. The inset regions A, B, C, D are mainly determined by shunt current, recombination current in diode space-charge region, diffusion current, diode diffusion current limited by series resistance, respectively. (Color figure online)

The dark current was fitted with Eq. 3 (single PN junction model); however, very limited information can be obtained except of three parameters (*m*, *J*_0_, *R*_s_) [7]. Furthermore, in the exponential coordinates, the dark current curve slope varies with the increase in voltage, reflecting different physical processes. This physical process cannot be reflected by Eq. 3 (single PN junction model). In fact, as shown in Fig. 4a, regions A, B, C of dark current is related to shunt current, recombination current and diffusion current, respectively. At last, above the built-in potential at about 1.2 V in region D, the effect of the recombination current is negligible, and the curve is determined only by the diffusion current, limited by the series resistance (*R*_s_) of the cell [14]. In order to more accurate quantitative analysis of the dark *J*–*V* characteristic in regions A, B, C in the perovskite solar cells, regardless of region D, Eq. 8 can be futherly deduced in dark (not consider *J*_L_, *R*_s_):

12 $$J = \frac{V}{{R_{\text{sh}} }} + J_{0} \left\{ {e^{{\frac{qV}{{\left( {m_{1} + m_{2} } \right)KT}}}} - 1} \right\}$$

In region A of Fig. 4a, the dark current is mainly affected by the shunt current under the small applied bias voltage. With the bias voltage increase, recombination current is much larger than diffusion current in dark *J*–*V* characteristics of planar PSCs, as shown in region B of Fig. 4a. The slope of region B is less than slope of region C, and the steep increment of the current results from a diffusion-dominated current [14]. The dark current–voltage characteristic is in a single exponential relationship in region B and region C, respectively.

In order to quantitative calculate diffusion current and recombination current, respectively, Eq. 12 is further rewritten by taking into account the heterojunction diffusion model [29, 30]:

13 $$J = \frac{V}{{R_{\text{sh}} }} + J_{\text{r}} \left\{ {e^{{\frac{{eV_{1} }}{{m_{{1{\text{r}}}} KT}}}} - 1} \right\} + J_{\text{d}} \left\{ {e^{{\frac{{eV_{1} }}{{m_{{1{\text{d}}}} KT}}}} - 1} \right\}$$

In Eq. 13, the first term is the shunt current corresponding to region A in Fig. 4a. The second term is the recombination current (Shockley–Read–Hall recombination), *m*_1r_ = 2. The third term is the diffusion current (including carrier directly recombination), *m*_1d_ = 1 [14, 36, 37]. According to Eqs. 9, 13 can be furtherly derived:

14 $$J = \frac{V}{{R_{\text{sh}} }} + J_{\text{r}} \left\{ {e^{{\frac{eV}{{m_{\text{r}} KT}}}} - 1} \right\} + J_{\text{d}} \left\{ {e^{{\frac{eV}{{m_{\text{d}} KT}}}} - 1} \right\}$$

In Eq. 14, *m*_r_ = *m*_1r_ + *m*_2r_ = 4, *m*_d_ = *m*_1d_ + *m*_2d_ = 2. The dark current in Fig. 4a can be fitted by Eq. 14. The calculation results of three parameters (*R*_sh_, *J*_r_, *J*_d_) are shown in Table 4.

**Table 4:** *R*_sh_, *J*_r_, *J*_d_ values derived from fitting dark current in Fig. 4a by using Eq. 14

MAI:MACl	*R*_sh_ (MΩ cm^2^)	*J*_r_ (mA cm^−2^)	*J*_d_ (mA cm^−2^)
50:0	12	1 × 10^−5^	5 × 10^−11^
50:2.5	16	9 × 10^−6^	3 × 10^−10^
50:5	20	8 × 10^−6^	5 × 10^−9^
50:7.5	19	8 × 10^−6^	3 × 10^−9^

We fit the dark current of inverted planar heterojunction PSCs in other literatures in order to further verify the formula based on double PN junction equivalent circuit (Fig. 1b) [8].

As shown in Table 5, the parameter *R*_sh_ of inverted planar PSCs with different hole-blocking layers is 1, 5, and 35 MΩ cm^2^. The recombination current (*J*_r_) of corresponding devices is 3 × 10^−6^, 1.5 × 10^−6^, and 5 × 10^−7^ mA cm^−2^. Both of them indicate that hole-blocking layer blocks hole injection into the diode, effectively reducing the shunt current and recombination current. Compared to the BCP, the device with PFN shows better hole-blocking property. The same conclusion obtained by comparing the dark current at − 100 mV as discussed in the literatures. Meanwhile, the devices without hole-blocking layer showed larger dark current under reverse bias, mainly due to the larger hole injection into the diode [8]. Moreover, the PFN enhanced electron injection and extraction, which can be verified by drastically increased diffusion current *J*_d_ (from 8 × 10^−10^ to 1 × 10^−9^ mA cm^−2^). This conclusion confirms the speculation in the literature: PFN improves the electron injection and extraction in PSC devices [8]. Therefore, the new model proposed in this study can be universal and effective to analyze carrier recombination and transportation.

**Table 5:** *R*_sh_, *J*_r_, *J*_d_ derived from fitting dark current of inverted planar PSCs in Fig. 4b

Hole-blocking layer	*R*_sh_ (MΩ cm^2^)	*J*_r_ (mA cm^−2^)	*J*_d_ (mA cm^−2^)
Without	1	3 × 10^−6^	8 × 10^−10^
BCP	5	1.5 × 10^−6^	4 × 10^−10^
PFN	35	5 × 10^−7^	1 × 10^−9^

## Conclusion

In conclusion, we built up a double PN junction equivalent circuit to fit *J*–*V* curves of P–I–N planar structure heterojunction PSCs. The new method focuses on the relationship between the diode ideal factor and the carrier recombination from the interface defects. By varying Cl^−^ content in the CH_3_NH_3_PbI_3_ perovskite film, we found that the value of m drastically diminished (decreased) with the perovskite film quality improvement. In order to quantitatively analyze the correlation mechanism of dark current under different bias voltages, a new equation based on the double PN junction equivalent circuit has been proposed to analyze the dark current–voltage curve. Consequently, carrier recombination and loss reduction could be reflected in *R*_sh_ and *J*_r_. The carrier transmission could be reflected on the parameter *J*_d_. Based on the double PN junction equivalent circuit, the *J*–*V* curve in light and in dark could be fitted, respectively, helping us analyze the working mechanism and improve the efficiency of planar PSCs.
